# *Pseudomonas guariconensis* Necrotizing Fasciitis, United Kingdom

**DOI:** 10.3201/eid3001.231192

**Published:** 2024-01

**Authors:** Edward J. Moseley, Jian Cheng Zhang, O. Martin Williams

**Affiliations:** UK Health Security Agency Specialist Microbiology and Laboratories, South West Region and Severn Infection Sciences, Bristol, UK (E.J. Moseley, J.C. Zhang, O.M. Williams);; University Hospitals Bristol and Weston NHS Foundation Trust, Bristol (E.J. Moseley, O.M. Williams);; Southmead Hospital, Bristol (J.C. Zhang)

**Keywords:** Pseudomonas guariconensis, bacteria, antimicrobial resistance, fasciitis, integrons, beta-lactamase, humans, United Kingdom

## Abstract

We describe a case of necrotizing fasciitis in the United Kingdom in which *Pseudomonas guariconensis* was isolated from multiple blood culture and tissue samples. The organism carried a Verona integron-encoded metallo-β-lactamase gene and evidence of decreased susceptibility to β-lactam antimicrobial agents. Clinicians should use caution when treating infection caused by this rare pathogen.

A 67-year-old man in the United Kingdom was seen in the emergency department for right lower leg pain and swelling with associated fevers lasting 24 hours. He reported a right heel blister had formed 1 week earlier, after he purchased new footwear. His medical history included obesity, hypertension, atrial fibrillation, and left ventricular systolic dysfunction. He was a former smoker and had a 40 pack-year history.

On examination, the patient appeared alert and comfortable. He was febrile (38.0°C), tachycardic (124 beats/min) in atrial fibrillation, and had a stable blood pressure (108/72 mm Hg). His respiratory rate was 20 breaths/min, and oxygen saturation was 92% on room air. He had right leg swelling and erythema, extending from the blister on his heel to his mid-calf. Blood test results showed leukocyte count was 15.03 × 10^9^ cells/L (reference range 4.0–11.0 cells/L), neutrophils 13.44 (reference 1.5–8.0) × 10^9^ cells/L, and lymphocytes 0.38 (reference 1.0–4.0) × 10^9^ cells/L. Bilirubin was 41 (reference <21) μmol/L, albumin 34 (reference 35–50) g/L, and C-reactive protein 20 (reference <6.0) mg/L.

The patient was started on intravenous flucloxacillin (1g 4×/d) for lower limb cellulitis. Aerobic blood culture samples at admission were positive at 11.5 hours’ incubation and cultures collected 8 hours after admission positive at 10.5 hours’ incubation by BD Bactec FX system (Becton Dickinson, https://www.bd.com). Gram stain from the samples showed gram-negative bacilli, resulting in an immediate change of therapy to intravenous amoxicillin/clavulanic acid (1.2 g 3×/d) and gentamicin (400 mg; 5 mg/kg based on patient’s ideal bodyweight). Direct extract from the first sample was tested by matrix-assisted laser desorption/ionization time-of-flight (MALDI-TOF) mass spectroscopy (Bruker Corporation, https://www.bruker.com), which identified *Pseudomonas guariconensis* with a score of 1.94 within 3 hours of the initial culture report. Growth on plates from the second blood culture was subsequently confirmed to be the same organism. Samples were sent to the UK Health Security Agency Antimicrobial Resistance and Healthcare Infection reference laboratory, which also confirmed *P. guariconensis* by MALDI-TOF mass spectroscopy with a score of 2.62.

The patient’s treatment was changed to intravenous piperacillin/tazobactam (4.5 g 4×/d); gentamicin was continued. On review, no local features of necrotizing fasciitis were observed, and his leg appeared improved. The patient reported that he had been applying several over-the-counter creams of uncertain age to his blister since it had developed. Attempts were made to recover the creams for culture but were unsuccessful.

Direct disk susceptibility testing was performed by using European Committee on Antimicrobial Susceptibility Testing (EUCAST) rapid antimicrobial susceptibility testing (AST) methodology, reading plates at 16–18 hours, which showed piperacillin/tazobactam susceptibility. Although unvalidated for this organism, AST suggested piperacillin/tazobactam susceptibility at increased exposure compared with EUCAST rapid AST breakpoints for *P. aeruginosa* and standard AST clinical breakpoints for *Pseudomonas* spp. (EUCAST criteria version 11.0), which was confirmed by standard EUCAST disk diffusion testing (*1*) from the second isolate. The patient’s gentamicin was stopped. However, he remained tachycardic and hypotensive.

Confirmatory AST performed by using the VITEK 2 system and software version 9.02 (bioMérieux, https://www.biomerieux.com) produced a piperacillin/tazobactam MIC of 64 mg/L and meropenem MIC of 4 mg/L (Table). Piperacillin/tazobactam MIC by gradient strip testing performed on the second isolate was increased at 16 mg/L, particularly close to the EUCAST breakpoint, and meropenem MIC was increased at 4 mg/L. The patient’s therapy was changed to 2 g intravenous ceftazidime (2 g 3×/d).

**Table:** Antimicrobial susceptibility data for blood culture isolates in a case of *Pseudomonas guariconensis* necrotizing fasciitis, United Kingdom*

Antimicrobial agent	Admission blood culture by ViTek 2† (MIC, mg/L)	8-h blood culture		Tissue culture by disc diffusion‡
Disc diffusion‡	Gradient strip (MIC, mg/L)
Ceftazidime	I (4)	I	ND	I
Ciprofloxacin	I (<0.25)	I	ND	I
Gentamicin	S (<1)	S	ND	S
Meropenem	I (4)	I	I (4)	I
Tobramycin	S (<1)	S	ND	S
Piperacillin/tazobactam	R (64)	I	I (16)	I
Amikacin	S (<2)	ND	ND	ND
Cefepime	I (2)	ND	ND	ND
Imipenem	I (1)	ND	ND	ND
Levofloxacin	I (0.5)	ND	ND	ND
Ticarcillin/clavulanic acid	R (>128)	ND	ND	ND

*All breakpoints are European Committee on Antimicrobial Susceptibility Testing (EUCAST) clinical breakpoints version 11.0, except gentamicin disk diffusion testing, for which EUCAST version 9.0 was used. I, susceptible, increased exposure; ND, not done; R, resistant; S, susceptible. †bioMérieux, https://www.biomerieux.com. ‡Using methods from EUCAST (*1*).

In-house multiplex PCR was performed using agarose gel electrophoresis for beta-lactamase genes (Appendix), based on previously published methodology (*2*–*5*). PCR detected a Verona integron-encoded metallo-β-lactamase enzyme, consistent with previously reported strains of this species (*6*,*7*).

The patient subsequently deteriorated and required inotropic and vasopressive support. He underwent above-knee amputation and debridement after fasciotomies, and exploration confirming necrotizing fasciitis. Tissue samples isolated pure growth *P. guariconensis*, and sensitivity testing by standard EUCAST disk methodology was consistent with previous samples (Table).

The patient remained in the critical care unit for 3 days and had high vasopressor requirements despite adequate antimicrobial drug therapy. He was deemed not stable for further surgery; life-sustaining treatment was withdrawn, and he died. 

*P. guariconensis* is a gram-negative, strictly aerobic, non–spore-forming, rod-shaped bacterium that is motile by means of 2 polar flagella, is oxidase and catalase positive, and is indole and aesculin negative. *P. guariconensis* was described in 2013, isolated from rhizospheric soil of *Vigna unguiculata* (L.) Walp. (the cowpea) in Guárico, Venezuela (*8*). Isolates of the same species producing novel carbapenemases have been reported from environmental samples taken in the Amazon Basin (*9*).

Reports of *P. guariconensis* human disease are rare; 1 case of infective endocarditis was reported in a patient with underlying lupus erythematosus (*10*). The rarity of reports likely reflects the recent description of the species and delays in updates to identification methodologies, such as MALDI-TOF databases. This case shows the pathogenic potential of *P. guariconensis* in an immunocompetent host and the degree of clinical suspicion required to exclude deep infection when isolating an unusual organism from a sterile site. 

Appendix Additional information on a case of *Pseudomonas guariconensis* necrotizing fasciitis, United Kingdom.
