# Supplementary material for: Pseudomonas guariconensis Necrotizing Fasciitis, United Kingdom
Source: Emerg Infect Dis. 2024 Jan;30(1):185–7. doi: 10.3201/eid3001.231192 (PMC10756353; doi:10.3201/eid3001.231192)
Supplement: Appendix — Additional information on a case of Pseudomonas guariconensis necrotizing fasciitis, United Kingdom. [file 23-1192-Techapp-s1.pdf]

Article DOI: https://doi.org/10.3201/eid3001.231192

EID cannot ensure accessibility for supplementary materials supplied by authors. Readers who have difficulty accessing supplementary content should contact the authors for assistance.

Pseudomonas guariconensis Necrotizing Fasciitis, United Kingdom

Appendix

Additional Methods

We performed an in-house multiplex PCR using agarose gel electrophoresis for beta-lactamase genes, including New Dehli metallo-β-lactamase (NDM), oxacillinase-type β-lactamase (OXA-48), *Klebsiella pneumoniae* carbapenemase (KPC), Verona integron-encoded metallo-β-lactamase (VIM), imipenemase-type metallo-β-lactamase (IMP), *Pseudomonas aeruginosa* extensive resistance (PER), Kyorin University Hospital metallo-β-lactamase (KHM), Dutch imipenemase (DIM), Triploi metallo-β-lactamase (TMB), and Adelaide imipenemase (AIM), based on previously published methodology (1–4).

References

1. Hidalgo L, Hopkins KL, Gutierrez B, Ovejero CM, Shukla S, Douthwaite S, et al. Association of the novel aminoglycoside resistance determinant RmtF with NDM carbapenemase in Enterobacteriaceae isolated in India and the UK. J Antimicrob Chemother. 2013;68:1543–50. PubMed https://doi.org/10.1093/jac/dkt078

2. Ellington MJ, Kistler J, Livermore DM, Woodford N. Multiplex PCR for rapid detection of genes encoding acquired metallo-beta-lactamases. J Antimicrob Chemother. 2006;59:321–2. PubMed https://doi.org/10.1093/jac/dkl481

3. Poirel L, Héritier C, Tolün V, Nordmann P. Emergence of oxacillinase-mediated resistance to imipenem in *Klebsiella pneumoniae.* Antimicrob Agents Chemother. 2004;48:15–22. PubMed https://doi.org/10.1128/AAC.48.1.15-22.2004

4. Yigit H, Queenan AM, Anderson GJ, Domenech-Sanchez A, Biddle JW, Steward CD, et al. Novel carbapenem-hydrolyzing β-lactamase, KPC-1, from a carbapenem-resistant strain of *Klebsiella pneumoniae.* Antimicrob Agents Chemother. 2001;45:1151–61. PubMed https://doi.org/10.1128/AAC.45.4.1151-1161.2001
